# A novel computer-aided diagnostic approach for detecting peripheral arterial disease in patients with diabetes

**DOI:** 10.1371/journal.pone.0199374

**Published:** 2018-06-21

**Authors:** Eva Elina Buschmann, Lulu Li, Michèle Brix, Andreas Zietzer, Philipp Hillmeister, Andreas Busjahn, Peter Bramlage, Ivo Buschmann

**Affiliations:** 1 Dept. for Cardiology, Center of Internal Medicine, Medical University Graz, Graz, Austria; 2 Dept. for Angiology, Center for Internal Medicine 1, Medical University Brandenburg (MHB), Brandenburg/ Havel, Germany; 3 Richard-Thoma Laboratories for Arteriogenesis, Center for Cardiovascular Research (CCR), Charité Universitaetsmedizin Berlin, Berlin, Germany; 4 Department of Physiology Charité Benjamin Franklin, Berlin, Germany; 5 Medizinische Klinik II, Universität Bonn, Bonn, Germany; 6 HealthTwiSt GmbH, Berlin, Germany; 7 Institute for Pharmacology and Preventive Medicine, Cloppenburg, Germany; Spectrum Health, UNITED STATES

## Abstract

Peripheral arterial disease (PAD) is an important manifestation of systemic atherosclerosis, with diabetes being one of its most significant risk factors. Owing to medial arterial calcification (MAC), the ankle–brachial index (ABI) is not always a reliable tool for detecting PAD. Arterial Doppler flow parameters, such as systolic maximal acceleration (ACCmax) and relative pulse slope index (RPSI), may serve as effective surrogates to detect stenosis-induced flow alteration. In the present study, ACCmax and RPSI were prospectively evaluated in 166 patients (304 arteries) with clinical suspicion of PAD, including 76 patients with and 90 patients without diabetes. In the overall sample, the sensitivity of ACCmax (69%) was superior to that of ABI (58%) and RPSI (56%). In patients with diabetes, the sensitivity of ACCmax (57%), ABI (56%) and RPSI (57%) were similar, though a parallel test taking both ACCmax and RPSI into account further increased sensitivity to 68%. The specificity (98%) and accuracy (78%) of ACCmax were superior to those of ABI (83% and 70%, respectively), as were the specificity (95%) and accuracy (77%) of RPSI in patients with diabetes. The diagnostic properties of ACCmax and RPSI were superior to those of ABI for detecting PAD in patients with diabetes. Our acceleration algorithm (Gefäßtachometer^®^) provides a rapid, safe, noninvasive tool for identifying PAD in patients with diabetes.

## Introduction

Peripheral arterial disease (PAD) is a chronic, slow-developing, atherosclerotic degenerative condition that results in the narrowing of peripheral arteries. Such progressive narrowing may result in ischemic pain, tissue damage, and eventual loss-of-limb when blood flow to the lower extremities fails to meet the metabolic demands of skeletal muscle [1]. Diabetes mellitus is one of the most significant risk factors for the development of PAD. In patients with diabetes, infra-popliteal arteries are often involved; this leads to progressive hemodynamic interference of arterial inflow, which then requires revascularization. Therefore, knowledge of an individual’s hemodynamic characteristics is indispensable for achieving adequate diagnosis and management. Moreover, compared to patients without diabetes, those with diabetes more commonly lack ischemic symptoms and suffer from medial arterial calcification (MAC). These factors complicate the process of reaching an accurate and timely diagnosis of interrupted inflow.

The ankle–brachial index (ABI) is a well-established parameter for non-invasive lower limb vascular screening that is recommended by all major guidelines [2–4]. A key advantage is that it is simple to measure without additional equipment by physicians and nurses and comes at low cost. An ABI of ≤0.90 has a high sensitivity and specificity for PAD [5], while an ABI ≥ 1.30 is indicative of MAC [6]. ABIs of ≤0.90 or ≥1.30 are also associated with an increased risk of major cardiovascular events, even going beyond traditional risk scores [7]. A definite disadvantage of the ABI is that a value of ≥1.30 is not an appropriate diagnostic tool for identifying PAD in the non-compressible calcified arteries that typify MAC.

In patients with diabetes, Doppler-derived maximal systolic acceleration (ACCmax) values could serve as a surrogate for detecting stenosis-induced deceleration in distal tibial arteries. This is a promising approach to identifying PAD independently of blood pressure measurements. Furthermore, by dividing the ACCmax by the mean velocity, an improved surrogate may be derived, referred to as the relative pulse slope index (RPSI). RPSI is significantly higher in arteries than in veins and has been shown to be a specific parameter for the detection of arterial flow patterns [8].

In the context of these findings, we aimed firstly to validate a computational approach for the determination of ACCmax and RPSI in patients with diabetes, and secondly to evaluate the diagnostic performance of each parameter for identifying PAD (using angiography as the diagnostic gold standard reference). We also aimed to explore the diagnostic value of the two parameters combined, and to challenge the idea that ABI is an adequate approach to the detection of PAD in patients with diabetes.

## Materials and methods

The study protocol was approved by the Charité ethics council (EA1/146/12) and was conducted in line with the Declaration of Helsinki and institutional ethics regulations. All patients provided their written informed consent to participate.

### Study populations

The current population is comprised of patients with suspected PAD who had been referred to a vascular laboratory to establish a diagnosis and those with established PAD that were attending a follow-up visit, screened between June 2011 and December 2014. To be included, patients had to be aged 50 years or older and have at least two of the following risk factors: hypertension, diabetes, dyslipidemia, and coronary artery disease (see section 2.5 for definitions). Exclusion criteria included lower limb disability, thrombosis within the last six months, severely reduced left ventricular ejection fraction (LVEF < 20%), arrhythmia (e.g. atrial fibrillation), and simultaneous participation in another clinical study.

ABI calculation (performed according to the standard protocol [9]) plus a duplex ultrasound of the pelvic and lower-limb arteries were carried out in all patients that were screened for the study. In each limb, the artery with the lowest perfusion pressure was taken for analysis. Those with a “normal” ABI of >0.90 for both limbs and without evidence of occlusive stenosis at duplex ultrasound (suspicion of PAD ruled out) did not undergo angiography. Those with an ABI of ≤0.90 or ≥1.30 and occlusive stenosis of >25% at ultrasound (clinical suspicion of PAD) were referred for angiography, unless a reference angiogram from within the last six months prior to enrolment had already been obtained. Patients who were diagnosed with atherosclerotic stenosis of >25% but who did not have an angiography available were excluded.

Patients were divided into two groups according to the presence/absence of diabetes, so as to assess the performance of the different diagnostic measures in these distinct patient populations. To these ends, each group was further subdivided into individuals with and without PAD.

### Ultrasound-derived ABI, ACCmax and RPSI

An ultrasound system (Philips HDX 11, Germany) with an L12-3 MHz transducer was used for all measurements throughout the study. ABI, ACCmax and RPSI were all assessed in one artery per limb following 10 minutes of rest, with measurements collected from the same surface projection of each artery (the region of interest was marked). Two measurements were taken within the same session to evaluate intra-measurement variance, separated by a 10-second interval. The ultrasound was performed by a single operator, who was blinded to the patient’s historical data but aware of the presence/absence of infra-popliteal PAD due to screening of the infragenual artery to identify the vessel of interest.

ACCmax was defined as the maximal slope of the velocity curve in the early systolic phase. Rather than visually determining the maximal slope or employing the commonly used diagnostic algorithms, pulsed-wave Doppler data were exported via a CE-certified and medical product-compliant audio output (CE 0086, SN 1078496). Curves were then investigated on a beat-by-beat basis using the previously published modified geometric method (MGM). For this purpose, the real-time velocity curve was computed with instantaneous maximal velocity values. By this means, the analog signal of acceleration could be obtained through the external differentiation of the velocity waveform, as first proposed by Sabbah *et al*. The accuracy of this technique was then improved by digitizing the ensemble-velocity curve based on the arithmetic average to reduce inter-beat variability. The accuracy and reproducibility of the digital values fell within ±5% of the values derived from the analog waveforms. The analysis program was initiated immediately upon data acquisition to assess its quality and to obtain a definitive value for ACCmax. As such, the operator remained blinded to this analysis and ACCmax was objectively determined from analogue data exported during the scan. Two additional blinded, independent operators reviewed this data.

By simultaneously computing the arithmetic average of flow velocities and by summarizing the obtained pulsatile curves to an ensemble velocity curve, the maximal slope of the ensemble curve was calculated as:

$$ACCmax=max\left(\frac{\Delta v}{\Delta t}\right)$$

and RPSI as:

$$RPSI=\frac{ACCmax}{Vmean}$$

where ACCmax is the maximum slope of the acceleration phase of the curve, Δ*v* is the velocity gradient, Δ*t* is the elapsed time, and Vmean is the mean velocity.

### ABI measurement

For each patient, the ABI for both sides was calculated as the ratio of the lowest of the two ankle blood pressure measurements to the highest of the brachial artery pressures [10, 11]. An ABI of ≤0.90 was classified as positive for PAD, an ABI of >0.90 to <1.30 as normal, and an ABI of ≥1.30 as indicative of MAC [7].

### Semi-quantitative angiography assessment and pulsed-wave Doppler flow measurement

**Fig 1:**
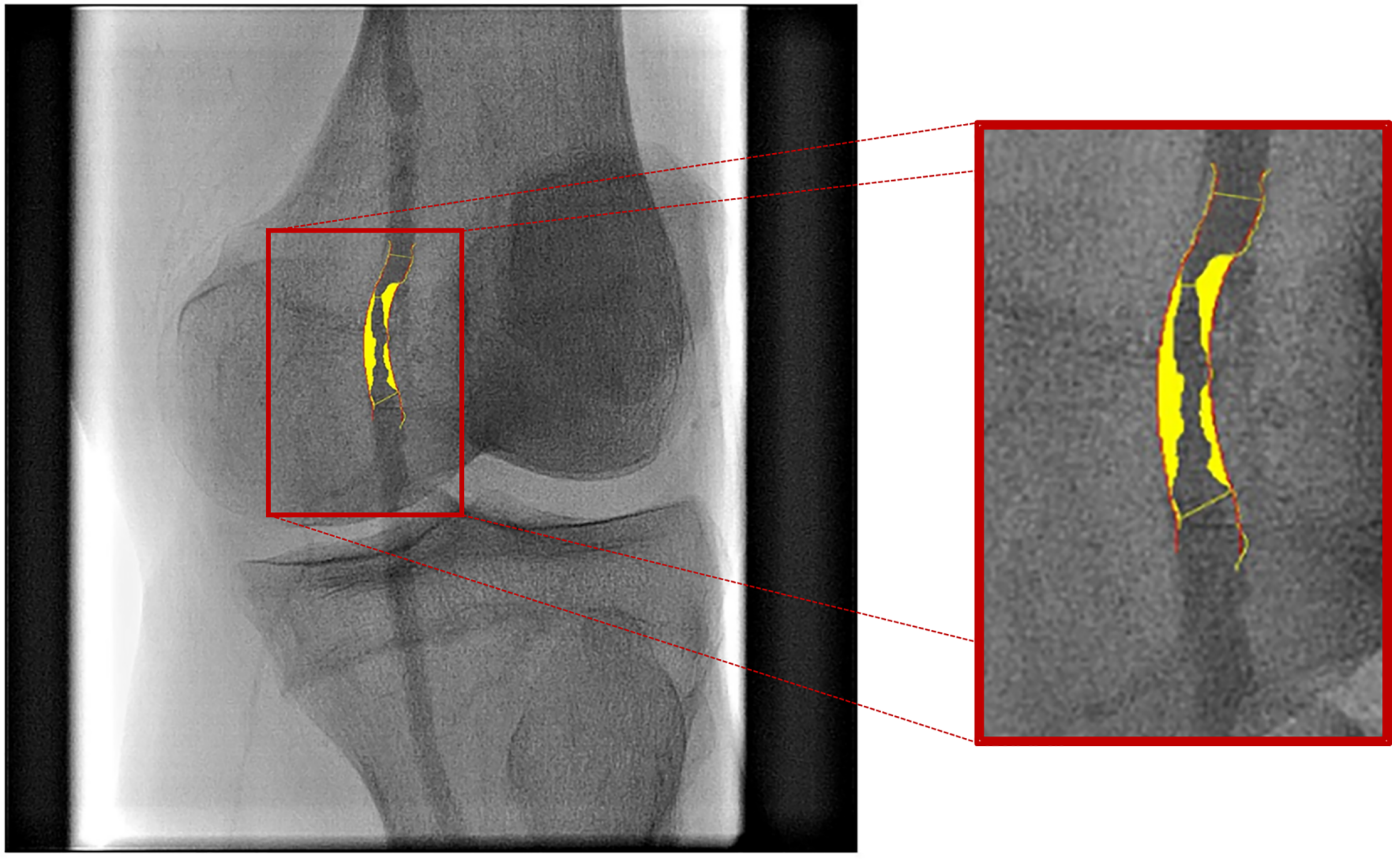
Example of quantitative angiography analysis. Red lines represent the reconstructed reference contours (positioned along the vessel segment), while the yellow area represents the plaque. In this particular angiographic case, the obstruction diameter was 1.17 mm, and the reference diameter at the narrowing was 3.46 mm, yielding a percentage diameter stenosis of 66%.

Angiography data were evaluated using quantitative analysis software (SDS, Sanders Data Systems, USA). Specific features of the SDS included 2-point user-defined path line (center-line) identification, arterial contour detection, and a reference vessel diameter. The minimal diameter within the lesion was used to calculate the percent stenosis relative to the diameter of the reference vessel (**Fig 1**). By this means, the lesions were located, and the degree of stenosis graded for each limb [9, 12]. The highest degree of stenosis was recorded as the maximum relevant stenosis.

**Fig 2:**
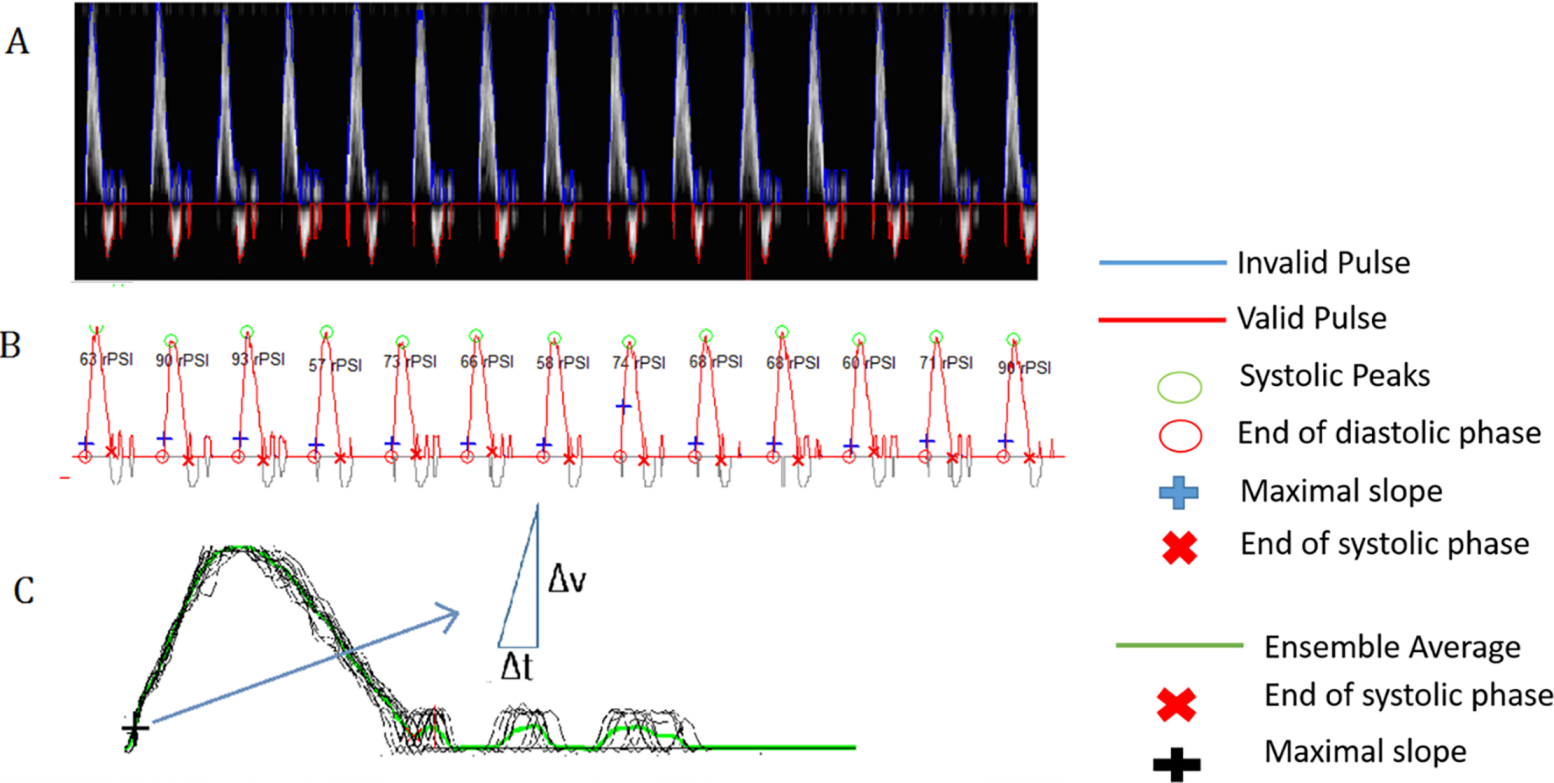
Example of triphasic waveforms measured in the normal distal tibial artery and schematic representation of the principle acceleration algorithm. A) Waveforms captured within 15 seconds in pulsed-wave mode. The blue line demonstrates the digital contour produced by the modified geometric method. B) Digitalized contoured curves are presented. The blue cross shows the maximal slope of the systolic phase. C) The ensemble of the velocity-curve is shown as a green line, where each point is based on the arithmetic average of the velocity values. The black cross shows the maximal slope of the systolic phase on the ensemble curve.

Doppler data were gathered at rest after 10 minutes of relaxation in the horizontal position. All tests were performed between 10 am and 4 pm in a lockable, artificially lit room with dimmed lights and a constant temperature between 22–23°C. Smoking and the consumption of beverages containing xanthines were prohibited during the 4 hours preceding data collection, with medication taken as usual. Waveform characteristics and flow velocities were recorded (**Fig 2**). The signals were categorized as physiological (I), fine monophasic (II), weak monophasic (III), or weak monophasic with diastolic forward flow (IV) [13, 14]. A weak monophasic signal was differentiated from a fine weak monophasic signal by a severely decreased peak flow velocity of <30 cm/s (**Panel A in S1 Fig**) and further categorized into severely decreased with/without diastolic forward flow (**Panel B in S1 Fig**). ACCmax, RPSI, and ABI values were compared between these four waveform subgroups. More information on the different Doppler types are available in Nelson et al. [15].

### Definitions

Hypertension was defined as systolic blood pressure (SBP) > 140 mmHg, diastolic blood pressure (DBP) > 90 mmHg, both of these, or use of antihypertensive medication. Diabetes was defined as use of anti-diabetic treatment or documented presence of the condition. Dyslipidemia was defined as use of lipid-lowering therapy. Cardiovascular disease (CVD) was defined as a history of myocardial infarction, acute coronary syndrome (or percutaneous coronary intervention), coronary artery bypass graft, or > 50% stenosis of the coronary artery confirmed by coronary angiogram. Chronic kidney disease (CKD) was defined as a moderate reduction in the glomerular filtration rate (30–59 ml/min/1.73 m^2^).

### Statistical analysis

Sample size calculation was based on the general assumptions of a significance level of 5% and a statistical power of 80%. We planned to test the sensitivities of RPSI (the novel parameter), ACCmax, and ABI (the standard parameter) for equivalence at a 95% confidence level. The number of measurements (i.e. number of limbs) necessary was estimated to be 73, equivalent to approximately 40 patients. We also planned to carry out comparisons between the sensitivities of RPSI and ACCmax using superiority tests. Assuming a drop-out rate of 5%, we estimated that 140 patients would be sufficient to reach the desired statistical power.

Numerical data were tested for normal distribution using Kolmogorov–Smirnov tests. Normally distributed data are presented as means with standard deviations, while non-normally distributed data are given as medians with interquartile ranges (IQR). As only RPSI was normally distributed, all comparisons of continuous variables were carried out using a Wilcoxon rank sum test to ensure comparability. Categorical variables are reported as absolute numbers and relative frequencies (%), with comparisons carried out using a Fisher's exact test.

The relationships between the diagnostic methods were explored graphically by linear regression and local polynomial regression fitting (LOESS). For quantification, Spearman rank correlation coefficients (rho) were computed.

To determine the diagnostic properties (sensitivity, specificity and accuracy) of the different diagnostic methods, hemodynamic measurements were subjected to receiver operating characteristic (ROC) analysis. The area under the curve (AUC) was computed and the threshold for diagnostic parameters set as per the Youden criterion, which maximizes the distance to the diagonal identity and thus determines the optimal balance between sensitivity and specificity. Quantitative angiography assessment was set as the diagnostic gold standard against which to evaluate the diagnostic performance of ACCmax, RPSI, and ABI. In this context, PAD was defined as >50% stenosis in one or more of the main limb arteries (*A iliaca communis*, *A iliaca externa*, *A*. *femoralis superficialis*, *A poplitea*, *A tibialis anterior*, *A tibialos posterior*, *or A fibularis*). For the purposes of the present study, >50% stenosis of the *A iliaca interna* or *A profunda femoris* was not considered diagnostic of PAD. This is because the study was designed to assess the value of different measurements for detecting impaired lower limb perfusion, which is largely unaffected by the hemodynamic impairment of these two arteries alone. Diagnostic performance was assessed on an individual-limb basis for the overall sample, as well as for patients with and without diabetes.

Two approaches to improving diagnostic performance by combining the different diagnostic methods were explored. The first employed a **linear logistic regression model**, which made use of data on RPSI, ACCmax, and ABI for each individual. We included all main effects without interaction. Sum of squares for analysis of deviances were computed according to the independent risk factor model (type 2 test). Based on the predicted probabilities of PAD, a **risk score** (probability of PAD) was computed and subjects with a score of >61% classified as testing positive. The second approach was a **parallel test**, which identified patients as having PAD when either their ACCmax and/or RPSI value was above the respective threshold. The diagnostic properties of the risk score and parallel test were then computed.

R language was employed for statistical analysis of diagnostic properties [16], carried out using the pROC package for ROC analysis [17]. A two-sided p-value of <0.05 was considered significant.

## Results

### Patient characteristics

More than 750 patients were screened for eligibility. A total of 166 patients (76 with diabetes and 90 without diabetes) were enrolled. In some patients it was only possible to obtain readings for one limb due to loss of limb or occlusion of the distal arteries. Thus, data for 304 distinct arteries (one per limb) were documented. In patients with diabetes, 158 arteries were analyzed, of which PAD was present in 75 cases and absent in 83 (**Table 1**). In patients without diabetes, 146 arteries were analyzed, of which PAD was present in 65 cases and absent in 81. All PAD limbs had undergone angiography (140 PAD limbs out of 241 angiography limbs).

**Table 1:** Baseline characteristics of study participants, stratified by presence/absence of diabetes.

	Patients with diabetes	Patients without diabetes	P-value
Number of participants	**76**	**90**	
Age [years]	70 (64, 79)	70 (62, 72)	>0.050
Gender [female]	24/76 (32)	37/90 (41)	0.051
CVD	62/76 (82)	58/90 (64)	0.001
CKD	19/76 (25)	26/90 (29)	>0.050
Hypertension	68/76 (89)	65/90 (72)	0.018
Number of limbs measured	**158**	**146**	
Limbs with PAD	75/158 (47%)	65/146 (45%)	0.646
ABI	1.04 ± 0.38	0.97 ± 0.32	0.060
RPSI	86 (51, 118)	80 (47, 123)	0.621
ACCmax	595 (408, 883)	584 (406, 862)	0.714

Data given as mean ± standard deviation, median [IQR], or n/N (%). CVD, cardiovascular disease; CKD, chronic kidney disease; PAD, peripheral artery disease; ABI, ankle–brachial index; RPSI, relative pulse slope index; ACCmax, systolic maximal acceleration. Patients with a “normal” ABI of >0.90 for both limbs and without evidence of occlusive stenosis at duplex ultrasound (suspicion of PAD ruled out) did not undergo angiography. All PAD limbs had undergone angiography.

No significant difference in the prevalence of chronic kidney disease (CKD) or PAD was found between patients with and without diabetes, while CVD and hypertension were more common in patients without diabetes.

After all relevant study-related data had been documented, three patients (two with diabetes and one without) with PAD underwent interventional revascularization. The index arteries were assessed after the procedure, during the 6-month follow-up. Two patients with diabetes took part in supervised cardiovascular training for 6 months. The three index arteries were also assessed twice.

### Semi-quantitative measurement of angiograms and Doppler waveform analysis

**Fig 3:**
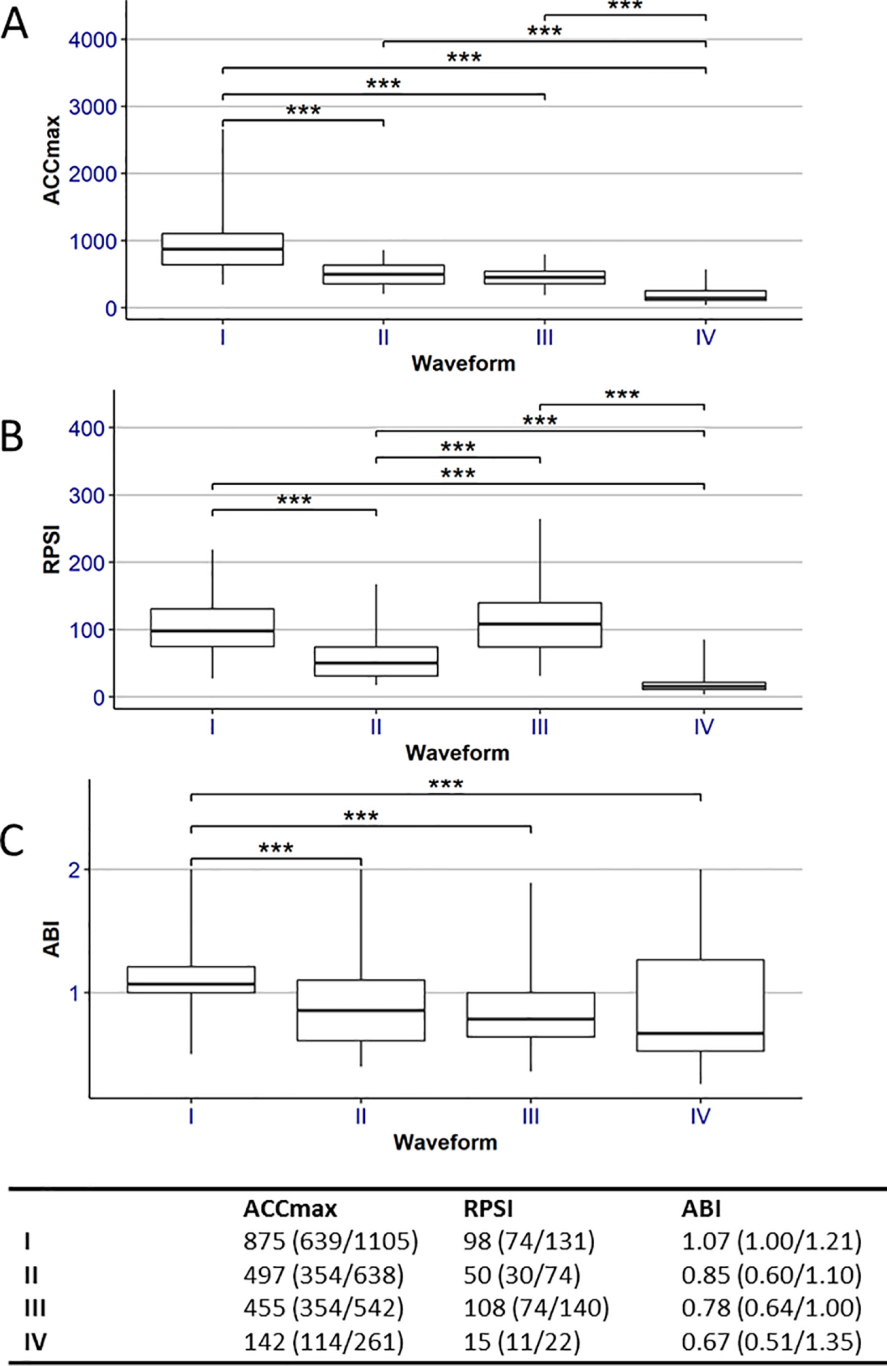
Comparison of hemodynamic values between different waveform subgroups from all patients. A) Comparison of ACCmax values between different waveform subgroups. B) Comparison of RPSI values between different waveform subgroups. C) Comparison of ABI values between different waveform subgroups. I: physiological; II: fine monophasic; III: weak monophasic; IV: weak monophasic with diastolic forward flow. ***p < 0.001.

Of the 304 arteries assessed, waveforms were physiological (I) in 150 cases, fine monophasic (II) in 57 cases, weak monophasic (III) in 63 cases, and weak monophasic complicated with diastolic forward flow in 34 cases. The median ACCmax, RPSI and ABI values per waveform subgroup are displayed in **Fig 3A, 3B and 3C,** respectively. No significant differences between waveform groups II–IV were observed for ABI values (**Fig 3C**). However, ACCmax values were significantly different between subgroups II and IV, as well as subgroups III and IV (**Fig 3A**). Furthermore, RPSI values were significantly different for every comparison between subgroups II, III and IV (**Fig 3B**; p < 0.001 for each comparison), indicating different stages of PAD.

### Discrimination of hemodynamic characteristics

#### Investigation of the distribution of ACCmax, RPSI and ABI in patients with and without diabetes

**Fig 4:**
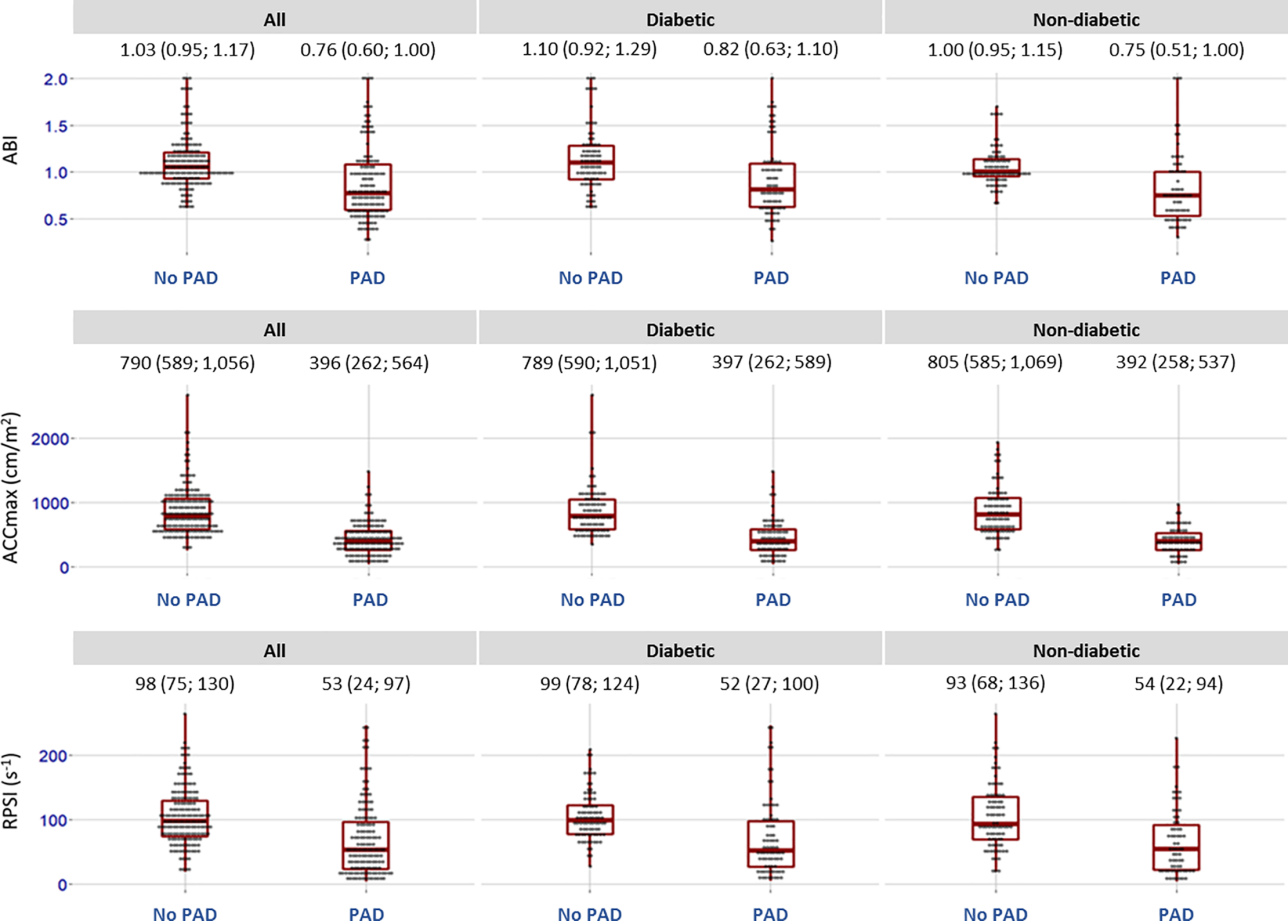
Comparison of ABI, ACCmax and RPSI values between the no-PAD and PAD group in all patients as well as those with and without diabetes. ABI: ankle-brachial index; ACCmax: systolic maximal acceleration, RPSI: relative pulse slope index. Data presented as medians with IQR, minimum and maximum values. Values stated are medians and IQRs. Medians were significantly different between PAD and no-PAD subgroups for all comparisons (p < 0.05).

In general, patients with PAD had lower ABI, ACCmax and RPSI values compared to patients without PAD, irrespective of whether or not they had diabetes; however, the values for PAD and no-PAD subgroups overlapped in all cases (**Fig 4**). The degree of this overlap was greatly reduced when using ACCmax. Indeed, while the central 50% of ABI and RPSI values (IQR, represented by the red boxes in **Fig 4**) overlapped substantially between PAD and no-PAD sub-groups, the IQRs for ACCmax values were discrete in every case.

#### Investigation of the relationship between ACCmax, RPSI and ABI in patients with and without diabetes

**Fig 5:**
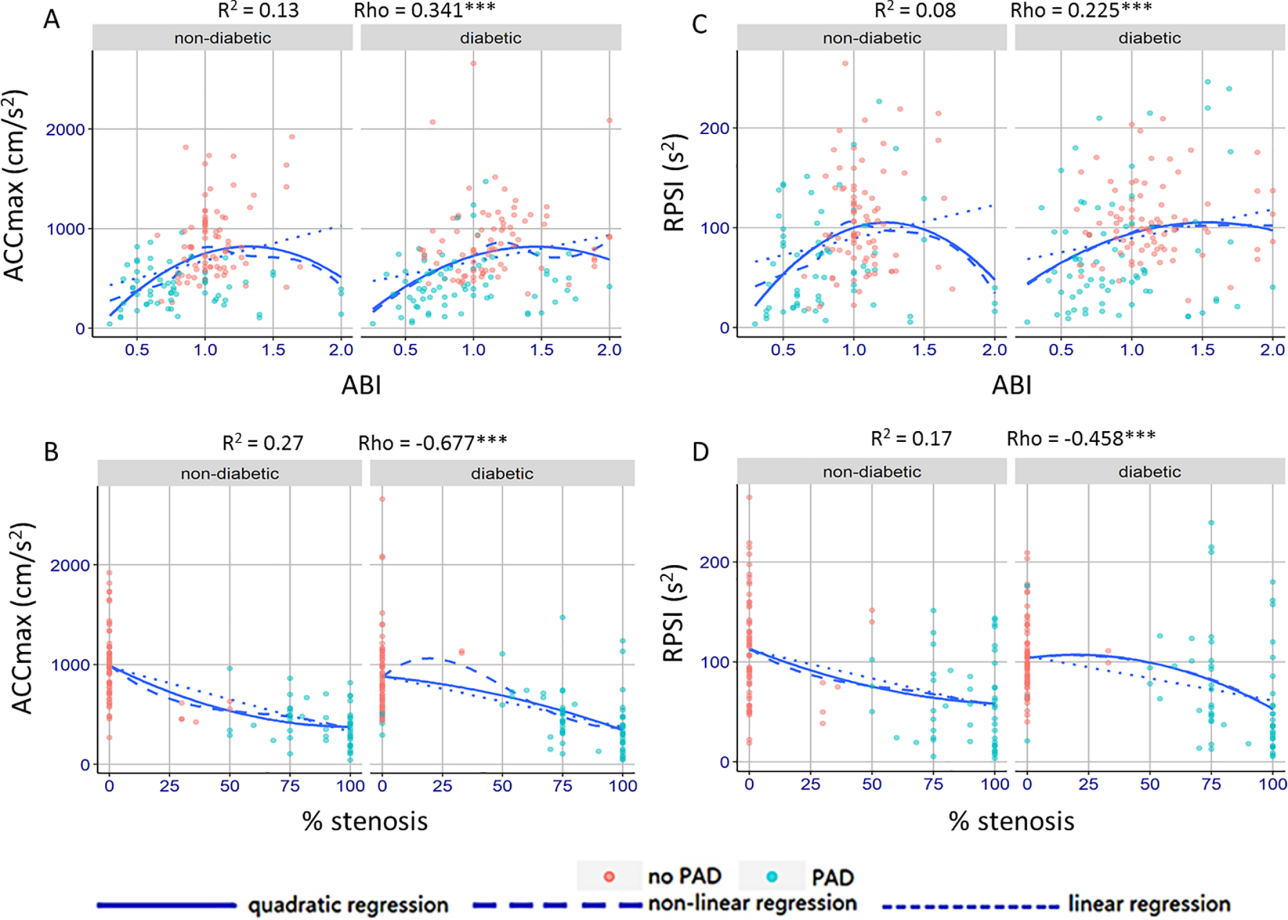
Correlation between novel parameters (ACCmax and RPSI) and ABI/percentage stenosis in patients with and without diabetes (quantitative evaluation). ABI, ankle-brachial index; ACCmax: systolic maximal acceleration, RPSI: relative pulse slope index; PAD, peripheral artery disease. Patients with PAD and MAC can be observed as blue points in the area of ABI ≤ 0.90, presenting with lower positive ACCmax and RPSI values. Patients without PAD but with MAC can be observed as red points in the area of ABI ≥ 1.30, presenting with extraordinarily high ACCmax values. *** p < 0.001.

ACCmax values decreased steadily alongside decreasing ABI values (positive correlation; R^2^ = 0.13; rho = 0.341; **Fig 5A**) and also decreased alongside an increasing degree of stenosis (negative correlation; R^2^ = 0.27; rho = -0.677; **Fig 5B**). There was a positive correlation between RPSI and ABI (R^2^ = 0.08; rho = 0.225; **Fig 5C**) and a negative correlation between RPSI and percentage stenosis (R^2^ = 0.17; rho = -0.458; **Fig 5D**). The correlation between ABI and percentage stenosis was also negative (R^2^ = 0.16; rho = -0.467) **(S2 Fig**).

### Diagnostic parameters of ACCmax, RPSI and ABI for diagnosing PAD in patients with and without diabetes

**Fig 6:**
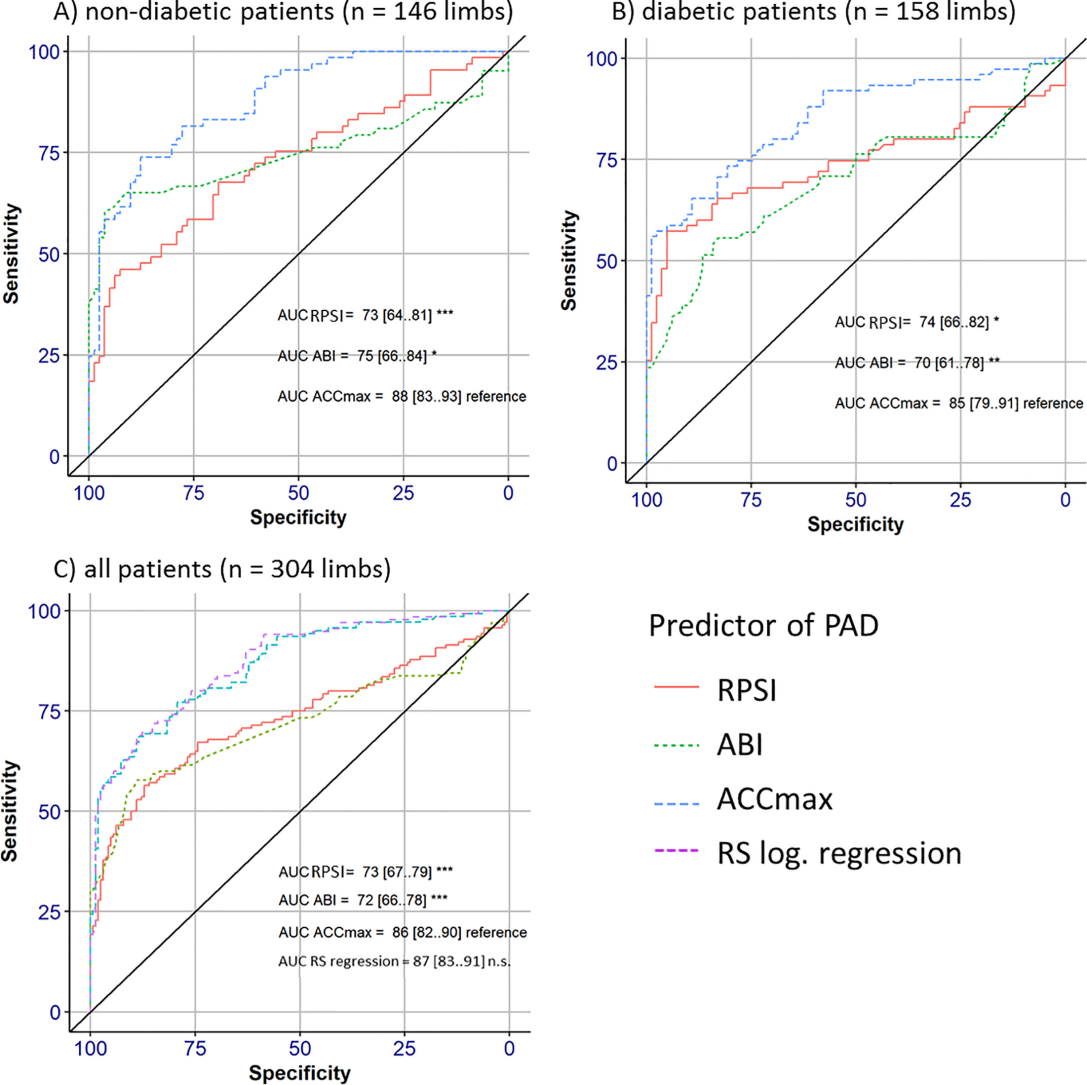
ROC curves for ACCmax, ABI and RPSI. AUC, area under the curve; ABI, ankle-brachial index; ACCmax: systolic maximal acceleration, RPSI: relative pulse slope index; RS, risk score. *p < 0.05; **p < 0.01; ***p < 0.001.

In all patients and in those with and without diabetes considered separately, the AUC for ACCmax was significantly larger than the AUCs for ABI and RPSI (**Fig 6**). In patients with diabetes specifically, the AUC for RPSI was also larger than that for ABI.

Based on the Youden Index, diagnostic thresholds **for the overall sample** were determined to be 503 cm/s^2^ for ACCmax, 58 s^-1^ for RPSI, and 0.83 for ABI (**Table 2**). While the specificities of the three measures using these thresholds were similar (ACCmax: 88%; RPSI: 87%; ABI: 89%), sensitivity (ACCmax: 69%; RPSI: 56%; ABI: 58%) and accuracy (ACCmax: 79%; RPSI: 73%; ABI: 75%) were both higher with ACCmax.

**Table 2:** ROC analysis for RPSI, ACCmax, and ABI.

	Threshold	Specificity (%)	Sensitivity (%)	Accuracy (%)
***All patients***				
ACCmax	503.00 cm/s^2^	88	69	79
RPSI	58.00 s^-1^	87	56	73
ABI	0.84	89	58	75
Risk score logistic regression*	0.61	89	69	80
***Patients without diabetes (with/without PAD)***				
ACCmax	498.00 cm/s^2^	88	74	82
RPSI	45.00 s^-1^	93	46	72
ABI	0.80	96	60	80
***Patients with diabetes (with/without PAD)***				
ACCmax	444.00 cm/s^2^	98	57	78
RPSI	58.00 s^-1^	95	57	77
ABI	0.88	83	56	70

AUC, area under the curve; ABI, ankle-brachial index; ACCmax: systolic maximal acceleration, RPSI: relative pulse slope index.

*Calculated for all patients only due to small n-numbers in diabetes-stratified subgroups.

Thresholds defined by the Youden criterion.

In **patients without diabetes**, the thresholds for ACCmax, RPSI and ABI were calculated to be 498 cm/s^2^, 45 s^-1^ and 0.80, respectively. The trends in sensitivity and accuracy observed for the overall sample were also applicable to this group, though specificity was highest with ABI (96%), followed by RPSI (93%) and ACCmax (88%).

In **patients with diabetes**, the thresholds were 444 cm/s^2^, 58 s^-1^, and 0.88 for ACCmax, RPSI and ABI, respectively. In these patients, the three measures had similar sensitivities (ACCmax: 57%; RPSI: 57%; ABI: 56%), though both specificity and accuracy were highest for ACCmax (specificity: 98%; accuracy: 78%), followed by RPSI (specificity: 95%; accuracy: 77%) and ABI (specificity: 83%; accuracy: 70%).

### Combination models of hemodynamic indices to identify PAD

#### Risk score logistic regression

In the logistic regression approach, which included RPSI, ACCmax, and ABI parameters, the significance level of ACCmax was <0.001 and that of ABI was <0.01, indicating that both were independently useful for the prediction of PAD. Subsequently, a new parameter based on ACCmax and ABI–the “risk score logistic regression”–was calculated for each subject using this model. Using the overall sample, ROC analysis revealed an AUC for the risk score logistic regression which was not significantly larger than that for ACCmax alone (**Fig 6C**). Furthermore, using a threshold of 0.61 (as determined by the maximum Youden Index), the specificity, sensitivity and accuracy of the risk score logistic regression were similar to those of ACCmax (**Table 2**).

#### Parallel test method

**Fig 7:**
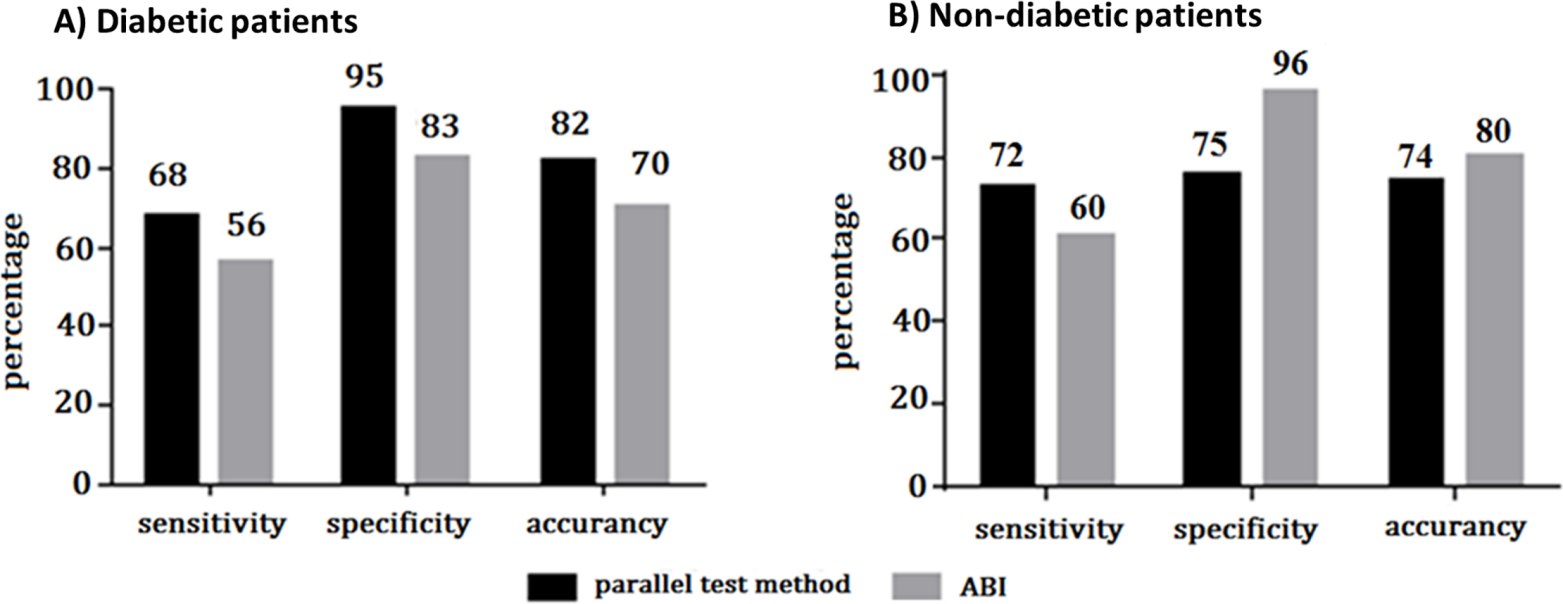
Comparison of the diagnostic sensitivity, specificity, and accuracy of the parallel test and ABI methods in patients with diabetes (limbs = 160) and in those without diabetes (limbs = 150). ABI, ankle-brachial index.

We implemented a parallel test method to detect PAD in all study participants with values above the calculated threshold for either ACCmax or RPSI. For the group of patients with diabetes, the parallel test achieved a specificity of 95%, which compared favorably with the value of 83% found for the ABI method (**Fig 7**). Importantly, the parallel test also improved sensitivity (68%) and accuracy (82%) when compared to ABI (56% and 70%), as well as ACCmax (57% and 78%) and RPSI (57% and 77%) considered separately.

## Discussion

The applicability of pulsed-wave Doppler for rapid, noninvasive detection of PAD is well-established, providing waveforms that vary from a normal peripheral artery waveform to those that are pathological. Our approach focused on the quantitative evaluation of such waveforms and the calculation of initial acceleration and mean velocity using a computer algorithm (Gefäßtachometer^®^) (**Fig 2**).

The difficulties associated with achieving a swift and reliable diagnosis of PAD in patients with diabetes is well-recognized. In 2006, a novel hemodynamic parameter, ACCmax, was proposed for the detection of renal arterial stenosis [18]. The diagnostic value of ACCmax was subsequently assessed in peripheral arteries by Tongeren *et al*. In spite of their promising data, ACCmax is not routinely used for the assessment of arterial stenosis, which is due to a number of limitations. Firstly, the data collection procedure is dependent on operator experience. Owing to the manual nature of the method, an irregular signal-to-noise ratio can result in inaccuracies. Secondly, the quantification of maximal acceleration is susceptible to artificial errors during the identification of the slope outline. While the error between visual and computed detection of ACCmax would be tolerable in a healthy population, it becomes relevant in the presence of vascular disease due to the low gradient of the slope. Thirdly, in the previous diagnostic study of ACCmax, ABI rather than the gold standard angiography was used to evaluate the performance of this novel parameter. Finally, the hemodynamic relevance is limited when only the initial systolic slope instead of the whole pulse wave is taken into account [19–21].

All aforementioned methodological pitfalls were addressed in our study. The first two limitations were addressed through applying a computerized algorithm (**Fig 2**). In this way, original Doppler signals were kept to a maximum, in contrast with the signal loss that occurs during the pre-processing of common commercial Doppler systems. As for assessments of ACCmax and RPSI, a standard computerized process minimizes artificial errors. Furthermore, the algorithm approach increases operator-friendliness, facilitating system use by nurses and physicians alike. As an additional advantage, it also results in an extremely rapid diagnostic tool (data acquisition time of <1 minute compared to >10 minutes for ABI assessment, including resting time) capable of simultaneous data analysis, and can be used prior to/during routine duplex investigation without extra cost- or time-expenditure.

To address the third of the aforementioned limitations, in agreement with current guidelines, angiography was used as the gold standard in our study. The close accord between our ACCmax values and those from the angiography, in combination with the superior results of the ROC analysis, favor the use of Doppler-derived ACCmax and RPSI for predicting the presence and degree of PAD. Similar to the study by Tongeren *et al*., we also observed a correlation between ACCmax and ABI (R^2^ = 0.13) and between RPSI and ABI (R^2^ = 0.08). The weak correlation between ACCmax and ABI indicated that these two parameters were independent and non-substituent diagnostic tools, with different hemodynamic aspects being observed. Furthermore, ACCmax and RPSI demonstrated diagnostic reliability when the ABI approach lost its function due to MAC (ABI ≥ 1.30).

In order to address the final limitation, taking the mean flow velocity of both the systolic and diastolic phase into account, the RPSI served as an additional parameter to ACCmax, even though RPSI cannot provide a diagnosis of PAD when used alone. For example, in two patients with similar ACCmax values but different Doppler waveform and mean flow velocity values, the slow and continuous forward flow could be identified via the low RPSI value (**Fig 3**). This characteristic was in agreement with our group’s finding in 2010 that RPSI could discriminate between the pulsatility of arteries and veins [8]. This was the first time that RPSI was validated in patients.

When comparing the diagnostic performance of ACCmax, RPSI, and ABI, **Figs 4 and 6** show a clear advantage of ACCmax over ABI. In particular, the overlap of values for PAD-positive and PAD-negative patients was considerably smaller for ACCmax measurements than for ABI measurements; ACCmax values falling within the IQR for PAD patients were entirely discrete from those for no-PAD patients, which was not the case for ABI. This difference was particularly striking in patients with diabetes. Furthermore, we observed the feasibility of RPSI for discriminating between different compensation levels and thus different stages of PAD in patients with and without diabetes. Similar staging was not possible using the ABI approach.

In line with the current literature, the performance of ABI and ACCmax was generally poorer in patients with diabetes [22, 23]. Regarding blood flow parameters such ACCmax, this may be explained by the abnormal metabolic state in individuals with diabetes having a profound effect on the structure and function of almost all vasculature (impaired micro-/macro-circulation of the arterial, venous and lymphatic vasculature) [24]. Such effects range from inflammation through to derangement of cellular components and alterations to blood cells. Accordingly, multiple hemostatic factors may be disturbed, potentially influencing ACCmax and decreasing its reliability as a marker of PAD. Conversely, the accuracy of RPSI appeared to be moderately greater in patients with diabetes compared to ABI and ACCmax. The superiority of RPSI in the diabetic population may be explained by the fact, that the RPSI takes the net forward blood flow (Vmean), even in the very same heart beat into account. The net forward blood flow reflects the nature (composition) of the inflow (cardiac output, hemodynamic properties of stenosis, pre-stenotic vasculature resistance) as well as the outflow of the arterial region of interest (post-stenotic vascular resistance, microcirculation). Given that in patients with diabetes affection of the microcirculation is more frequent compared to patients without diabetes this might be one admissable supposition. However, the reason for this remains unclear and merits further exploration.

The fundamental goal of the study was to determine whether patients were correctly assigned to the PAD or non-PAD group using our diagnostic approach. Thus, we hypothesized that a more accurate prediction could be achieved by combining the advantages of ACCmax and RPSI. The parallel test method confirmed our assumption by achieving a high specificity of 95% and improving sensitivity to 68% in patients with diabetes (**Fig 7A**). While it must be noted that this sensitivity is not ideal for a diagnostic test, it appears to be superior to that of ABI, which up until now has been employed as a reliable technique for detecting PAD. As for the patients without diabetes, even though the parallel test showed lower specificity than the ABI approach, use of ACCmax or RPSI individually may provide an additional advantage, with no need to combine the approaches (**Fig 7B**). In summary, while the parallel test could be employed in patients with diabetes to detect PAD, ACCmax or RPSI alone may have value in patients without diabetes, which needs to be explored in further trials and clinical practice. Furthermore, RPSI could provide the additional benefit of allowing estimation of hemodynamic compensation status, thus enabling the different stages of PAD to be discerned in patients both with and without diabetes.

Angiography has commonly been recognized as the gold standard for detecting PAD. Therefore, ACCmax values, RPSI values, and ABI values were all included in the correlation analysis along with the percentage stenosis identified using angiography, with ACCmax demonstrating the best correlation. However, even though angiography is able to show the exact anatomic location of the stenosis, hemodynamic assessment is not possible using this method. Therefore, we measured the ACCmax and RPSI at the distal tibial arteries around the ankle, providing a hemodynamic evaluation that was independent of the location of stenosis. The correlation between ACCmax and the percentage stenosis was moderate (R^2^ = 0.27), because in some individuals, these parameters were inversely associated. For instance, the observed monophasic waveforms could result from one hemodynamically relevant stenosis or multiple non-significant atherosclerotic lesions, since blood flow waves attenuated gradually to a monophasic pattern, with the second and third wave components being significantly diminished. On the other hand, due to collateral perfusion, proximal occlusion or stenosis of the femoral artery could develop into a triphasic signal on a Doppler examination of distal tibial arteries. This highlights the significance of evaluating blood perfusion from the distal tibial arteries, since net functional forward perfusion is a factor.

We observed cases of pathological flow complicated with MAC in our study, that is, arteries which were proven to be affected by PAD through angiography but that demonstrated false higher ABI values, (both ≥ 1.30 and 0.90–1.30) (**Fig 5**). Not surprisingly, evaluation of internal flow through measurement of ACCmax showed reliable performance in the cases displaying false higher ABI values. Therefore, it appears that ACCmax could identify PAD among the patients with a high risk of MAC. We also observed arterial flow under pure MAC, that is, arteries which were deemed to be negative for PAD through angiography but demonstrated higher ABI values of ≥1.30. Interestingly, the ACCmax values were extraordinarily high for such arteries, reaching greater than 1000 cm/s^2^ in some cases (**Fig 5**). Owing to the limited sample size, we were not able to perform a statistical evaluation of the collected data. However, our analysis showed that these astonishingly high values of ACCmax could help detect MAC. This can be reasonably explained based on the mathematical equation of ACCmax and its mathematical correlation with pulse wave velocity (PWV), which is the well-accepted validation approach for MAC [25, 26].

$$\begin{array}{l} \mathrm{According}\;\mathrm{to}\;\mathrm{Newton}’s\;\mathrm{second}\;\mathrm{law}\;\mathrm{of}\;\mathrm{motion}:\;F=m*a\\ \mathrm{and}\;\mathrm{Ohm}’s\;\mathrm{law}:\;F=\frac{\Delta p}{R}\\ \mathrm{we}\;\mathrm{could}\;\mathrm{develop}:\;a=\frac{\Delta p}{R*m} \end{array}$$

where Δ*p* is the local blood pressure gradient, *R* is the local blood resistance, m is the mass of local blood, and *a* is the blood acceleration. Thus, for a given blood resistance and given blood mass, acceleration is derived from the instantaneous maximal derivative of arterial pressure (Δ*p*).

Similar to the ABI, another frequently reported parameter for characterizing the condition of peripheral arteries is the toe–brachial index (TBI), which is determined more distally at the level of the big toes. Since digital arteries are less likely to be affected by MAC, TBI is assumed to be more sensitive than ABI for detecting PAD among patients with diabetes; however, its performance is still disputed [22, 27]. Indeed, occlusive arterial disease and concomitant MAC can result in a severely reduced effective net perfusion pressure in the toe, meaning that TBI is also unreliable [28].

### Limitations

Firstly, the use of two limbs from one patient may raise concerns regarding the dependence of observations and potential for type one error. Furthermore, treating limbs as independent samples may have reduced the statistical power. However, since the hemodynamic and perfusion pressure of each limb is different, we considered it appropriate to treat each limb as an individual entity. Secondly, as the ABI alone as well as in combination with duplex ultrasound determination of PAD status is not perfect, some limbs with PAD may have gone undetected. This would lead to a slight overestimation of the RPSI detection rate. Thirdly, ACCmax and RPSI were collected using a commercial ultrasound system, which requires much less professional knowledge of sonography than normal sonography techniques. We found abnormal higher ACCmax values in suspicious MAC populations, however we failed to observe reliable TBI values, and we did not apply the PWV method. Therefore, in our group’s forthcoming large-scale clinical study, the correlation analysis between ACCmax and PWV will be adjusted to improve the accuracy and impact of the conclusions. The post-hoc decision to stratify patients by the presence/absence of diabetes may be perceived as a limitation of the analyses.

## Conclusion

The present study offers evidence for the validity of a novel method for PAD diagnosis, involving digital translation of Doppler signals and computer-assisted calculation of hemodynamics. The approach appears to have the potential to improve diagnostic efficiency and accuracy, and should be considered for incorporation into future clinical strategies for identifying PAD, especially in patients with diabetes.

## Supporting information

S1 Fig Two types of weak monophasic waveforms corresponding to different stages of limb ischemia. A) a weak monophasic waveform with a peak velocity of approximately 30 cm/s. B) a weak monophasic waveform with a peak velocity of 30 cm/s and continuous forward flow, which is absent in Figure A in S1 Fig.
(TIF)

S2 Fig Correlation between ABI and percentage stenosis in all patients. ABI, ankle-brachial index; PAD, peripheral artery disease. *** p < 0.001.
(TIF)
