# Supplementary figures and images for: A novel computer-aided diagnostic approach for detecting peripheral arterial disease in patients with diabetes
Source: PLoS One. 2018 Jun 21;13(6):e0199374. doi: 10.1371/journal.pone.0199374 (PMC6013098; doi:10.1371/journal.pone.0199374)

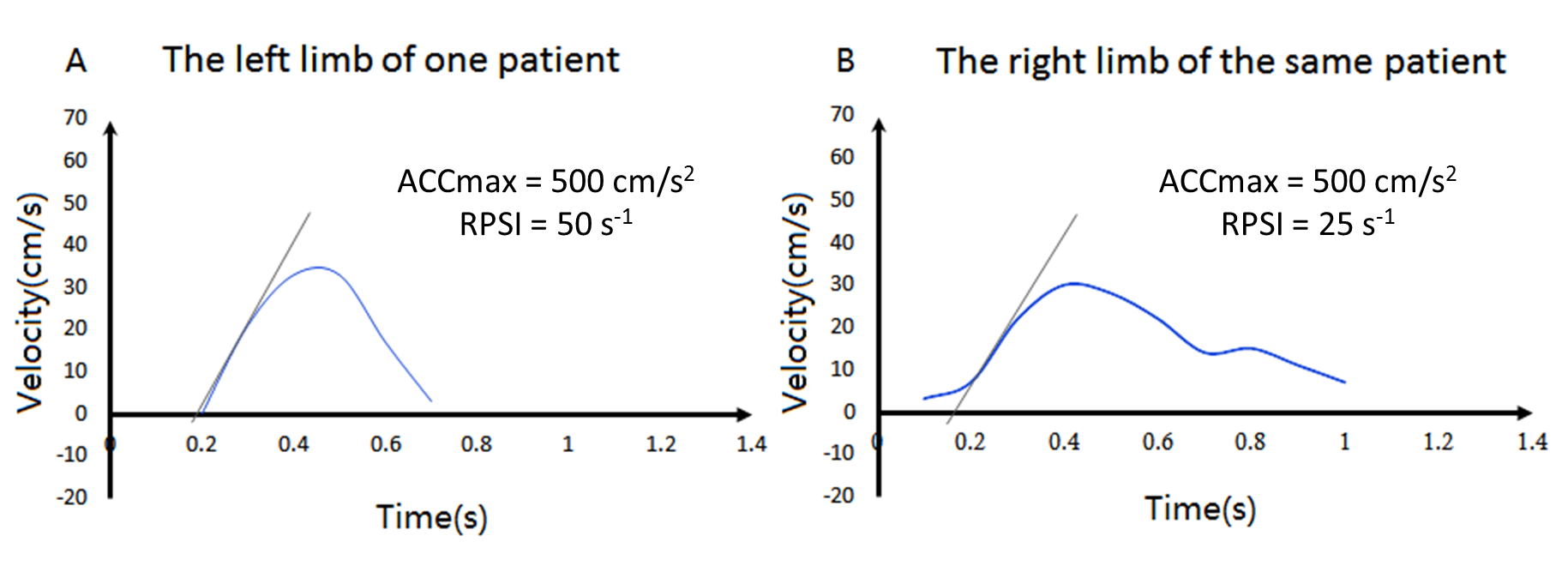

Supplement: S1 Fig — A) a weak monophasic waveform with a peak velocity of approximately 30 cm/s. B) a weak monophasic waveform with a peak velocity of 30 cm/s and continuous forward flow, which is absent in Figure A in S1 Fig. (TIF) [file pone.0199374.s001.tif]

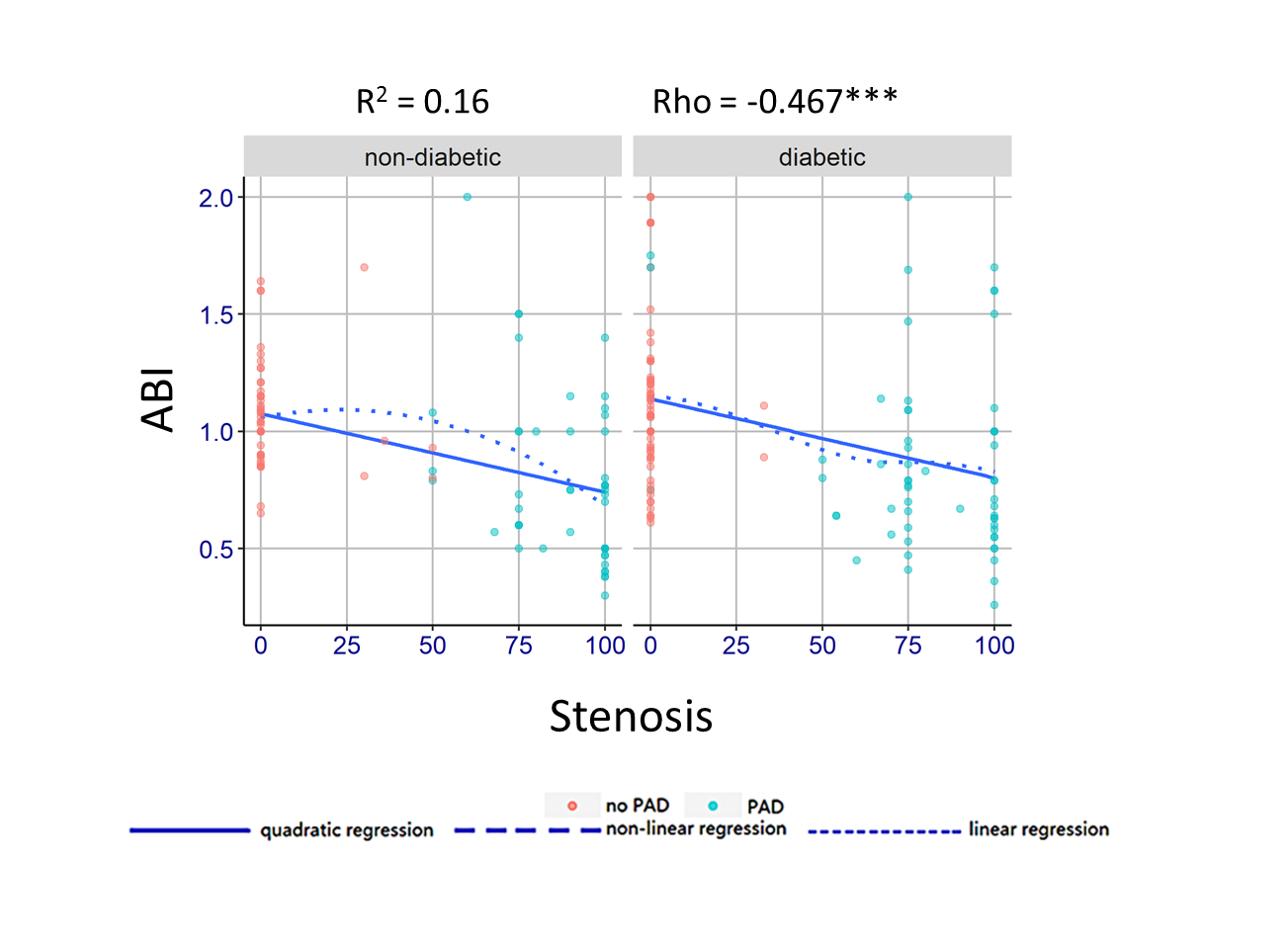

Supplement: S2 Fig — ABI, ankle-brachial index; PAD, peripheral artery disease. *** p < 0.001. (TIF) [file pone.0199374.s002.tif]
